# Germline BAP1 Mutation in a Family With Multi-Generational Meningioma With Rhabdoid Features: A Case Series and Literature Review

**DOI:** 10.3389/fonc.2021.721712

**Published:** 2021-08-24

**Authors:** Rahul N. Prasad, Ulysses G. Gardner, Alexander Yaney, Daniel M. Prevedello, Daniel C. Koboldt, Diana L. Thomas, Elaine R. Mardis, Joshua D. Palmer

**Affiliations:** ^1^Department of Radiation Oncology, The Ohio State University Comprehensive Cancer Center-Arthur G. James Cancer Hospital and Richard J. Solove Research Institute, Columbus, OH, United States; ^2^Boonshoft School of Medicine, Wright State University, Dayton, OH, United States; ^3^Department of Neurosurgery, The Ohio State University Comprehensive Cancer Center-Arthur G. James Cancer Hospital and Richard J. Solove Research Institute, Columbus, OH, United States; ^4^The Steve and Cindy Rasmussen Institute for Genomic Medicine, Nationwide Children’s Hospital, Columbus, OH, United States; ^5^Department of Pathology, The Ohio State University Comprehensive Cancer Center-Arthur G. James Cancer Hospital and Richard J. Solove Research Institute, Columbus, OH, United States

**Keywords:** rhabdoid meningioma, meningioma with rhabdoid features, familial *BAP1* tumor predisposition syndrome, biallelic *BAP1* inactivation, adjuvant radiation therapy, cancer screening, germline genetic testing, tumor sequencing

## Abstract

Meningioma is the most common primary brain tumor, and recurrence risk increases with increasing WHO Grade from I to III. Rhabdoid meningiomas are a subset of WHO Grade III tumors with rhabdoid cells, a high proliferation index, and other malignant features that follow an aggressive clinical course. Some meningiomas with rhabdoid features either only focally or without other malignant features are classified as lower grade yet still recur early. Recently, inactivating mutations in the tumor suppressor gene *BAP1* have been associated with poorer prognosis in rhabdoid meningioma and meningioma with rhabdoid features, and germline mutations have been linked to a hereditary tumor predisposition syndrome (TPDS) predisposing patients primarily to melanoma and mesothelioma. We present the first report of a familial *BAP1* inactivating mutation identified after multiple generations of a family presented with meningiomas with rhabdoid features instead of with previously described *BAP1* loss-associated malignancies. A 24-year-old female presented with a Grade II meningioma with rhabdoid and papillary features treated with subtotal resection, adjuvant external beam radiation therapy, and salvage gamma knife radiosurgery six years later. Around that time, her mother presented with a meningioma with rhabdoid and papillary features managed with resection and adjuvant radiation therapy. Germline testing was positive for a pathogenic *BAP1* mutation in both patients. Sequencing of both tumors demonstrated biallelic *BAP1* inactivation *via* the combination of germline *BAP1* mutation and either loss of heterozygosity or somatic mutation. No additional mutations implicated in oncogenesis were noted from either patient’s germline or tumor sequencing, suggesting that the inactivation of *BAP1* was responsible for pathogenesis. These cases demonstrate the importance of routine *BAP1* tumor testing in meningioma with rhabdoid features regardless of grade, germline testing for patients with *BAP1* inactivated tumors, and tailored cancer screening in this population.

## Introduction

Meningioma is the most common primary brain tumor, and recurrence risk increases with increasing WHO Grade from I to III (1). Rhabdoid meningiomas are a subset of WHO grade III tumors that predominantly consist of rhabdoid cells similar to other rhabdoid tumors (such as atypical teratoid rhabdoid tumor) and have a high proliferation index and other histologic features of malignancy (2, 3). These cases usually follow an aggressive clinical course, so traditional grading has excellent prognostic value. On the other hand, some meningiomas show rhabdoid features only focally and/or lack other features of malignancy (high mitotic rate, brain invasion, necrosis, macronucleoli, sheet-like growth, and hypercellularity). The WHO suggests these tumors be graded as usual with an added descriptor of “with rhaboid features”. These cases are thought to be less aggressive but closer follow-up may be warranted as notably some behave aggressively (2, 3).

Recently, inactivating mutations in the tumor suppressor gene *BAP1* that codes for the breast cancer (BRCA)1-associated protein, an important member of many vital pathways including DNA damage signaling and repair (4), have been associated with significantly decreased time to recurrence in patients with rhabdoid meningioma and meningioma with rhabdoid features (5, 6). Patients with a familial germline mutation in *BAP1* display a hereditary tumor predisposition syndrome (TPDS) leading to early onset malignancy – most frequently melanoma, mesothelioma, and renal cell carcinoma (6–9). Prior retrospective analyses and case series have discovered familial *BAP1* TPDS after patients presented with these malignancies, and in some cases, close relatives were later found to have meningiomas. However, to our best knowledge, we present the first report of an inherited *BAP1* inactivating mutation identified after several generations of a family presented with meningioma with rhabdoid features instead of with previously documented *BAP1* loss-associated cancers (7–9). We review these patients’ diagnoses, molecular profiling results, and management in the context of the literature to demonstrate the importance of *BAP1* tumor testing in meningioma with rhabdoid features regardless of grade, germline testing for patients with *BAP1* inactivated tumors, and tailored cancer screening in this population. Informed consent was obtained from both patients, and the institutional review board approved this study.

## Case Description

**Figure 1:**
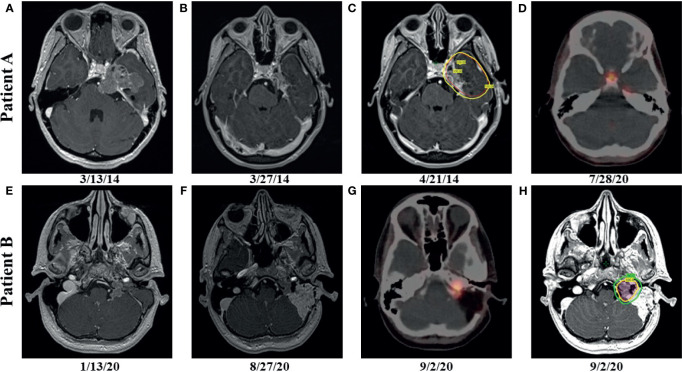
Patient A: preoperative, axial T1 postcontrast weighted magnetic resonance (MRI) imaging showing enhancing disease **(A)**; postoperative, axial T1 postcontrast weighted MRI imaging showing enhancing, residual disease **(B)**; radiation therapy (RT) planning using volumetric arc therapy (VMAT) resulted in excellent coverage of the planning target volume (PTV) (red) by the 100% isodose line (yellow) corresponding to 5940 cGy **(C)**; follow up gallium-68 dotatate positron emission tomography (PET) after more than 6 years showing hypermetabolic, recurrent disease in the left tentorial leaflet and physiologic uptake in the pituitary **(D)**; Patient B: preoperative, axial T1 postcontrast weighted MRI imaging showing enhancing disease **(E)**; postoperative, axial T1 postcontrast weighted MRI imaging showing enhancing, residual disease **(F)**; postoperative PET showing hypermetabolic, residual disease **(G)**; RT planning using VMAT resulted in excellent coverage of the 6000 cGy (red) and 5400 cGy (blue) PTVs by the 100% (yellow) and 90% (green) isodose lines, respectively **(H)**.

**Figure 2:**
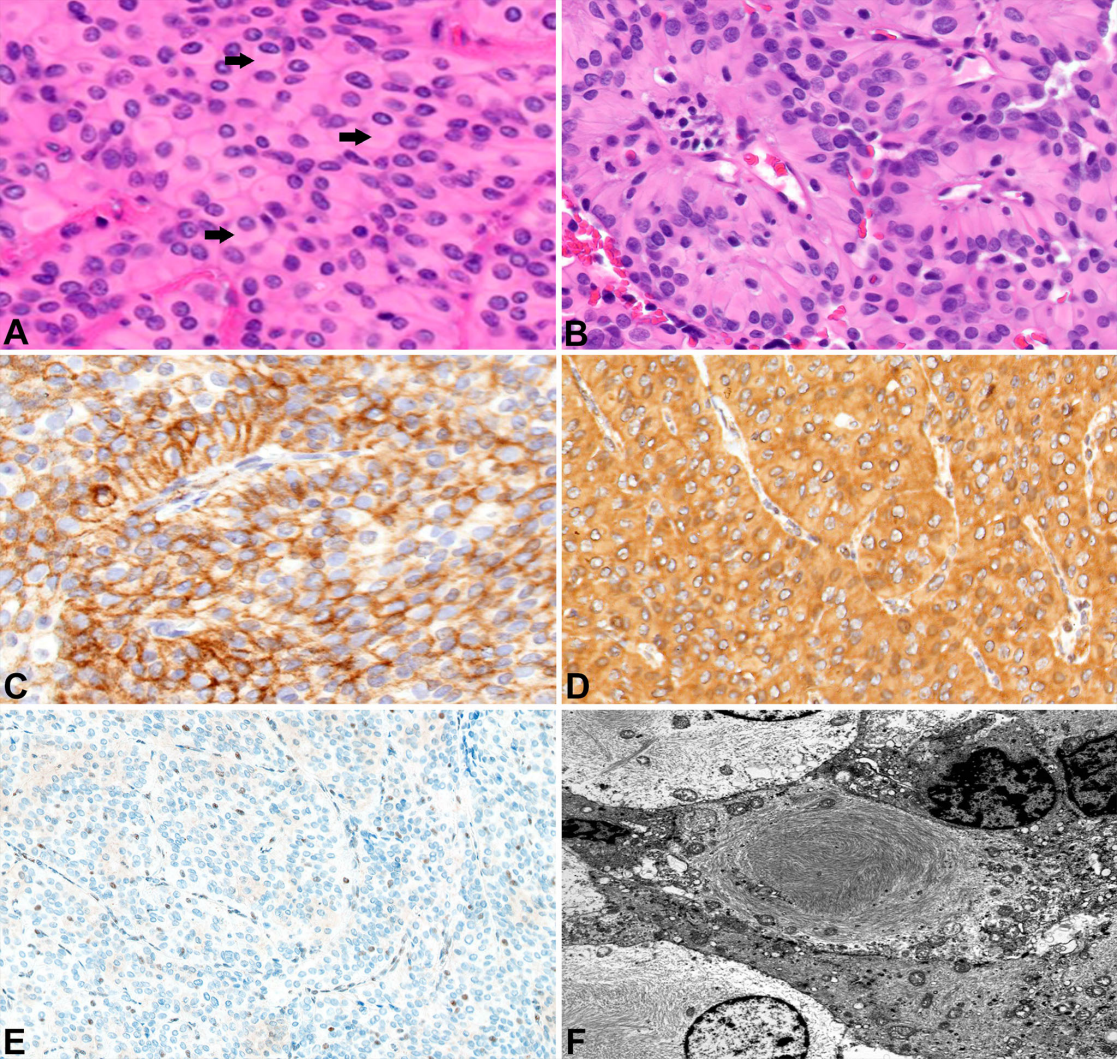
Surgical pathology for Patient A H&E stained sections show numerous rhabdoid tumor cells with cytoplasmic hypereosinophilic inclusions (arrows) (400x) **(A)**. Papillary features are also readily apparent (200x) **(B)**. EMA highlights the tumor cells (200x) **(C)**. The tumors cells label for vimentin (200x) **(D)**. There is loss of BAP-1 immunoreactivity in the tumor cells with appropriate internal positive controls (endothelial cells) (100x) **(E)**. Ultrastructural studies revealed cytoplasmic whorls of intermediate filaments **(F)**.

A 24-year-old female (Patient A) presented with decreased left facial sensation, blurry vision, left-sided frontal headaches, and pain with mastication of 3 months’ duration refractory to symptom-directed medical management. Physical exam revealed left masticator weakness, diminished sensation in the left V1-V3 distribution, and a decreased left-sided corneal reflex but was otherwise unremarkable. **Figures 1A–D** depicts a timeline for this patient presentation. Magnetic resonance imaging (MRI) of the brain with contrast revealed an infiltrating, contrast enhancing, roughly 5-cm mass centered in the left parasellar region with compression of the left temporal lobe, cavernous sinus, pons, and trigeminal nerve (**Figure 1A**). The patient underwent a left orbital frontal craniotomy with middle cranial fossa resection resulting in subtotal resection of the tumor. Pathologic evaluation revealed a meningothelial tumor composed of polygonal cells with mildly pleomorphic oval nuclei, abundant eosinophilic cytoplasm, and conspicuous cell borders. Many of the tumor cells showed eccentrically placed nuclei and round cytoplasmic inclusions of hypereosinophilic material consistent with rhabdoid morphology (**Figure 2A**). A significant component of papillary architecture was also present (**Figure 2B**). Mitoses were counted at 3 per 10 high power fields. The tumor cells showed diffuse immunoreactivity for EMA (**Figure 2C**) and vimentin (**Figure 2D**). There was complete loss of BAP-1 immunoreactivity in the tumor cells (**Figure 2E**) with appropriate internal positive controls (endothelial cells, inflammatory cells, etc.). The tumor cells showed focal staining for cytokeratin AE1/AE3. GFAP, S100, desmin, Melan-A, and HMB45 were negative. The Ki-67 proliferation index was 15%. Ultrastructural studies (electron microscopy) confirmed the presence of cytoplasmic whorls of intermediate filaments in many of the tumor cells (**Figure 2F**). Despite the rhabdoid and papillary features, in the absence of other malignant features (increased mitoses, necrosis, brain invasion, etc.), a diagnosis of atypical meningioma, WHO grade II, was ultimately made. Postoperative MRI brain demonstrated significant surgical decompression with residual enhancement along the left petroclinoid ligament and region of the cavernous sinus (**Figure 1B**).

Because of the subtotal resection of Grade II disease, adjuvant radiation therapy was recommended to 5940 cGy in 33 daily fractions. The patient was simulated in the supine position with an aquaplast mask for head and neck immobilization. To delineate treatment volumes, preoperative and postoperative T1-weighted axial postcontrast MRI imaging was fused to the computed tomography (CT) simulation scan. A preoperative gross tumor volume (GTV) was created to ensure that the clinical target volume (CTV) consisting of the residual contrast enhancing disease and postoperative bed appropriately covered the extent of the macroscopic and potential microscopic disease. An anisotropic expansion of 0-8 mm from the CTV was used to create a planning target volume (PTV) accounting for daily setup uncertainty while respecting dose constraints to the optic structures and brainstem (max dose less than or equal to 5400 cGy) (**Figure 1C**). Treatment was delivered using volumetric modulated arc therapy (VMAT) with 4 six MV arcs. RT was well tolerated with no significant adverse effects. The patient did well initially, but noted increasing headaches of several months duration over 6 years after resection. MRI brain with contrast showed increased nodular enhancement along the anterior margin of the left tentorial leaflet correlating with increased uptake on gallium-68 dotatate positron emission tomography (PET) imaging (**Figure 1D**). She underwent gamma knife radiosurgery to the recurrent disease to 22 Gy prescribed to the 50% isodose line.

**Figure 3:**
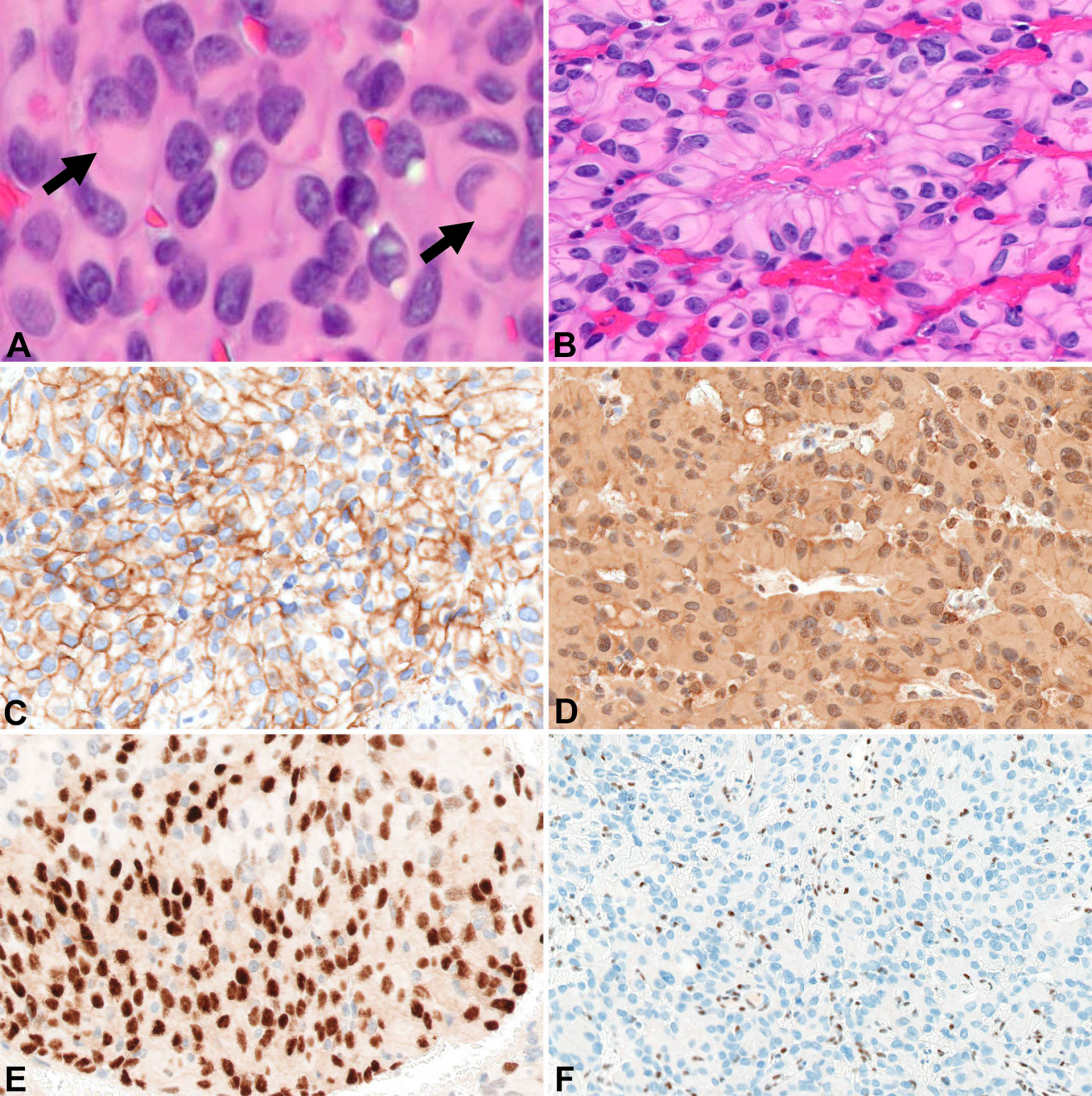
Surgical pathology for Patient B H&E stained sections show areas of rhabdoid tumor cells with cytoplasmic hypereosinophilic inclusions (arrows) (400x) **(A)**. Papillary arrangement of tumor cells (200x) **(B)**. EMA highlights the tumor cells (200x) **(C)**. Tumor cells are diffusely positive for vimentin (200x) **(D)**. Focal immunoreactivity for progesterone receptor in the tumor cells (200x) **(E)**. Diffuse loss of BAP-1 immunoreactivity in the tumor cells with appropriate internal controls (100x) **(F)**.

Around the same time, the 57-yo mother (Patient B) of Patient A presented with a 1-year history of left-sided, progressive hearing loss and pulsatile tinnitus refractory to symptom-directed medical management. On physical exam, she displayed left-sided conducting hearing loss without other abnormalities. **Figures 1E–H** depicts a timeline for this patient presentation. MRI Brain with contrast revealed a roughly 2.6 cm, enhancing left skull base mass involving the cerebellar-medullary angle cistern and centered around the jugular fossa with a tympanic component protruding into the middle ear cavity (**Figure 1E**). A left middle ear biopsy was consistent with meningioma with positive staining for EMA. She underwent a left tympanoplasty mastoidectomy resulting in subtotal resection of the tumor. Pathologic evaluation confirmed a meningioma with nearly identical appearance to the tumor of Patient A. Rhabdoid features were noted including eccentrically placed nuclei and abundant eosinophilic cytoplasm (**Figure 3A**). Papillary architecture with epithelioid cells arranged in perivascular papillae was also noted (**Figure 3B**). Mitoses were very rare (less than 1 per 10 high power fields). In support of the diagnosis, the tumor cells were immunoreactive for EMA (**Figure 3C**), vimentin (**Figure 3D**), and progesterone receptor (patchy; **Figure 3E**). The tumor cells showed diffuse loss of immunoreactivity for BAP-1 (**Figure 3F**). The tumor cells were also focally positive for S100, calponin, AE1/3, MNF116, CAM 5.2, CD138, SMA, and desmin. SHDHB, CD138, CD99, Melan-A, HMB-45, synaptophysin, and nuclear STAT6 were negative. Interestingly, SSTR2 immunostain was negative despite previous reports describing expression in most meningiomas including Grade II and III meningiomas (10, 11). The Ki-67 proliferation index was 10%. The tumor displayed infrequent mitoses (1 per 10 per HPF) and otherwise lacked anaplastic features. Postoperative MRI revealed residual tumor projecting into the cerebellomedullary angle, extending into the cerebellomedullary cistern and left jugular foramen, and tracking along the carotid sheath (**Figure 1F**) with corresponding increased uptake on gallium-68 dotatate PET (**Figure 1G**).

Because of the rhabdoid features potentially portending higher risk of recurrence, adjuvant radiation therapy to 6000 cGy in 30 fractions was recommended after the subtotal resection. The patient was simulated in the supine position with an aquaplast mask for head and neck immobilization. The postoperative PET as well as T1-weighted axial postcontrast and T2 flair MRI sequences were fused to the CT simulation scan and used to delineate a GTV encompassing the residual tumor. The GTV was expanded 0.3 cm to create a CTV and another 0.3 cm to create a PTV that was prescribed 5400 cGy in 30 fractions. A higher dose PTV receiving 6000 cGy in 30 fractions *via* a simultaneous integrated boost was created using a 0.2 cm expansion from the GTV that was then cropped off the left cranial nerves VII and VIII. The brainstem was constrained to a max dose of less than 5400 cGy, and the left cochlea and fifth, seventh, and eighth cranial nerves were to receive a mean dose of less than 5000 cGy. Treatment was delivered *via* VMAT with 3 six MV arcs (**Figure 1H**) and was well tolerated with no significant adverse events.

Due to the strong family history of meningioma with rhabdoid features and patient age at presentation, germline genetic testing was recommended. The daughter tested positive for a pathogenic *BAP1* variant (NM_004656.4:c.1777C>T, (p.Gln593*) with no other cancer susceptibility loci found mutated (CancerNext-Expanded, Ambry Genetics) (12, 13). Her mother subsequently tested positive for the same germline mutation. The c.1777C>T variant maps to exon 14 of *BAP1* and is predicted to introduce a nonsense change at amino acid 593. It has been reported as pathogenic by multiple clinical laboratories (ClinVar ID: 422219). The premature termination resulting from this variant would remove the BRCA1-binding domain (residues 594-657) (14). A previous study of a family with autosomal dominant, early-onset melanocytic neoplasms demonstrated that tumors which are biallelic for this variant show no BAP1 protein expression by immunohistochemistry. These findings suggest a loss-of-function effect mediated by nonsense-mediated mRNA decay (15).

**Figure 4:**
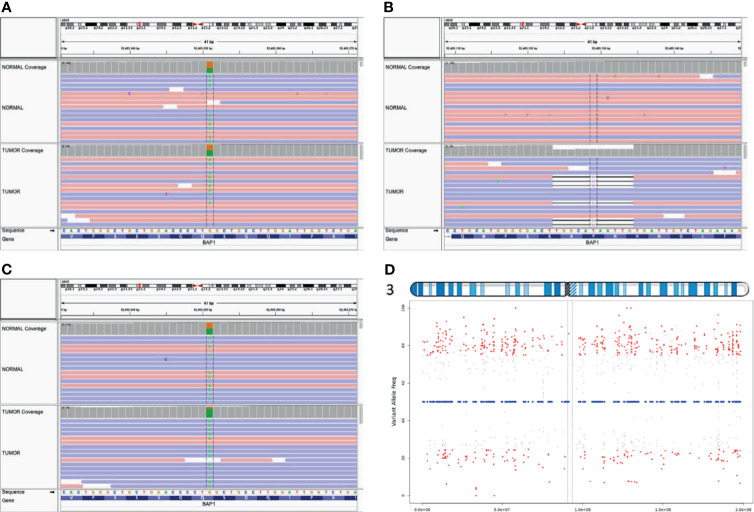
Biallelic inactivation of BAP1 by differing mechanisms. Aligned sequence data from Patient A show the pathogenic BAP1 variant is heterozygous in the germline (top track) and tumor (bottom track) **(A)**; the second hit is a somatic frameshift mutation **(B)**. In contrast, aligned sequence data from Patient B **(C)** show the germline variant approaching homozygosity in the tumor **(D)**. VarScan tumor allele frequency plot for heterozygous germline variants on chromosome 3 of Patient B indicates chromosome-level LOH across chromosome 3.

We submitted our interpretation of the variant to the ClinVar database [Accession: SCV001748998.1 (Submitted: Jul 09, 2021)]. A family history of kidney cancer at age of 52 in Patient B’s sister was discovered as well as lung cancer in patient B’s father (**Supplementary Figure 1**). No family history of malignancy was noted on Patient A’s father’s side of the family. We performed exome capture (IDT xGen Exome Research Panel v2.0 enhanced with the xGenCNV Backbone Panel and Cancer spike-in) and sequencing (NovaSeq6000) on tumor DNA and matched peripheral blood mononuclear cells from both mother and daughter (**Supplementary Document 1**). Analyses of these data supported the previous finding of germline *BAP1* (p.Gln593*) mutation in both tumors. The second inactivating somatic mutation in *BAP1* in Patient A’s tumor occurred through an 11-base pair frameshift mutation NM_004656.4:c.1092_1102del (p.His364GlnfsTer30) consistent with a loss of function *BAP1* variant (**Figures 4A, B**). By contrast, Patient B’s tumor exhibited LOH across the entirety of chromosome 3 including the second *BAP1* allele (**Figures 4C, D**). Patient A’s tumor had an additional 3 somatic coding mutations, and Patient B’s displayed an additional 18 somatic coding mutations (**Supplementary Table 1**). However, none of these mutations occurred in known cancer genes or in genes associated with meningioma (12) besides a missense somatic mutation in PIK3CA in the tumor of patient B. Mutations in PIK3CA have been previously associated with meningioma (16–18). This raises the possibility for genetic cooperation with BAP1-driven pathogenicity in tumor B, but this particular mutation has not been previously noted to be pathogenic.

At present, more than 7 years post- resection, Patient A has no clinical or radiographic evidence of newly recurrent or progressive disease. Nine months post-resection, Patient B displays no clinical or radiographic evidence of disease.

## Discussion

We have highlighted the first account of a familial *BAP1* inactivating mutation identified after multiple generations of a family presented with meningioma with rhabdoid features instead of presenting with previously described malignancies (4, 7–9, 19). *BAP1* codes for the (BRCA)1-associated protein which is integral to many cellular pathways including DNA damage signaling and repair, and inactivating mutations of this tumor suppressor gene are oncogenic (4). Patients with germline mutations display a TPDS phenotype leading to early onset malignancy, most frequently melanoma, mesothelioma, and renal cell carcinoma (4, 6–9, 19). Prior case series have documented inherited germline mutations after patients presented with these cancers (7–9). One report identified a germline *BAP1* truncating mutation, c.799 C>T (p.Gln267*), in a patient presenting with uveal melanoma leading to biallelic inactivation of *BAP1* with associated loss of function in this patient’s tumor as well as in a lung adenocarcinoma and meningioma in 2 additional relatives (7). Another case series highlighted a family presenting with multiple mesotheliomas and melanocytic tumors found to have an inherited germline *BAP1* mutation (c.1948T>A (p.Tyr646*); two of the family members developed meningiomas in addition to their mesotheliomas (8). A larger series documented several families presenting with melanoma and mesothelioma secondary to an inherited *BAP1* (p.Asp404*) germline truncating mutation, and one case of papillary meningioma was identified by autopsy (9). These authors incidentally noted several cases of meningioma in patients with germline *BAP1* mutations, but our case series is unique in identifying the presence of a familial *BAP1* mutation directly through patient presentation with meningioma with rhabdoid features rather than mesothelioma, uveal melanoma, or cutaneous malignancy. One case of pediatric rhabdoid meningioma in a patient found to have a germline *BAP1* mutation has been previously reported, but the mutation may have been sporadic as no cancers were reported in the patient’s siblings or parents’ generation, genetic testing was not conducted for any family members, and no other meningiomas were identified in this family (20).

A detailed family history was unremarkable for cancer other than rapidly fatal cases of kidney cancer in Patient B’s sister at the age of 52 and lung cancer in Patient B’s father at age 59. Patient B’s father had no known risk factors associated with lung cancer. Given the relatively young age at which these fatal cancers were diagnosed, it is plausible that one or both of these deceased family members carried an undiagnosed germline *BAP1* mutation. Regardless, our case series provide further support for the need for referral for genetic testing for patients presenting with *BAP1* mutated rhabdoid meningioma. A recent genetic analysis and literature review suggested germline testing for patients with 2 more or *BAP1* TPDS associated tumors, a single tumor with unusually young age at presentation, or a family history (21). Due to the rarity of this condition, prospective experience is not available to guide screening guidelines for patients with a germline mutation. However, referral for annual dermatologic and ophthalmological screening should be considered due to retrospective data showing a high risk of uveal and cutaneous melanoma (6, 21). Even young family members should consider genetic testing as uveal and ocular melanomas may present as early as in the teenage years (6, 21). Screening renal ultrasound, MRI, or urinalysis for renal cell carcinoma could also be considered (21), as these patients are prone to renal cell carcinoma and *BAP1* mutated renal tumors appear to grow at a faster rate than kidney cancers driven by other mutations such as in von Hippel-Lindau disease (19).

Recently, inactivating *BAP1* mutations have been linked to significantly reduced time to recurrence in both Grade III rhabdoid and lower grade meningioma with rhabdoid features (26 months *versus* 116 months, p < 0.001, hazard ratio 12.89) (5, 6). Characteristic features of a Grade III rhabdoid meningioma include rhabdoid components in combination with malignant features such as high mitotic rate, brain invasion, necrosis, macronucleoli, sheet-like growth, or hypercellularity. These grade III meningiomas tend to behave with an aggressiveness consistent with their high grade. In contrast, outcomes for lower grade meningiomas with rhabdoid features are mixed (2, 3). Tumors receive this designation if either rhabdoid components are only focal or the previously noted aggressive features are lacking. Although some of these lower grade tumors are less aggressive (2, 3), an analysis by Shankar et al. noted that a subset with BAP1 inactivation recurred far earlier (5, 6). Despite not meeting the full criteria for Grade III rhabdoid meningioma, both patients’ meningioma with rhabdoid features in our study displayed biallelic *BAP1* inactivation *via* an inherited germline *BAP1* (p.Gln593*) mutation and biallelic inactivation by different somatic mechanisms. Neither tumor contained mutations in additional genes associated with meningioma (such as *NF2* and *AKT1) (* 6) or malignancy, strongly implicating *BAP1* inactivation in disease pathogenesis. Patient A had a relatively aggressive disease course consistent with previously reported experiences in rhabdoid meningioma. Her *BAP1* mutated meningioma recurred after roughly 6 years, or well before the median time to recurrence expected for a *BAP1* wild type rhabdoid meningioma as reported by Shankar et al. (5). Although Patient B has no evidence of recurrence 9 months after resection, further follow up is needed. Meningiomas with rhabdoid histopathologic features appear to encompass a diverse genetic spectrum, and *BAP1* function may be just as important, if not more so, than morphology. At this time, *BAP1* inactivation is not routinely tested for in rhabdoid meningioma let alone meningioma with rhabdoid features (6). Thus, neither patient was screened for malignancies at the time of diagnosis with meningioma. Fortunately, neither patient has yet developed a cancer despite an 85-100% lifetime risk (6, 21). However, upfront tumor testing for *BAP1* inactivation may have aided with prognostication and introduction of timely screening for melanoma and other malignancy.

Besides the relatively limited follow up for Patient B, additional limitations of this case series include that germline genetic testing for asymptomatic family members was not available, and surgical pathology was not accessible to pursue tumor sequencing for family members previously deceased secondary to malignancy. Regardless, we present the first report of a familial *BAP1* inactivation TPDS identified after multiple generations of a family presented with meningioma with rhabdoid features instead of presenting with malignancies. Inactivating *BAP1* mutations have been associated with inferior outcomes in rhabdoid meningioma and meningioma with rhabdoid features, and routine tumor testing for this mutation may aid prognostication in lower grade tumors with rhabdoid features. Because germline mutations produce a TPDS, genetic testing should be offered to patients found to have *BAP1* inactivated tumors. Despite a lack prospective evidence to inform guidelines for screening for malignancy in this population, surveillance for ocular and cutaneous melanoma for patients carrying this germline mutation should be considered based upon elevated rates of disease in retrospective series. Given the paucity of available data to guide management of this rare condition, additional work is needed to determine the optimal diagnostic and management strategy for *BAP1* mutated meningioma with rhabdoid features and develop consensus guidelines for screening patients with inactivating germline mutations for malignancy.

## Data Availability Statement

The original contributions presented in the study are included in the article/**Supplementary Material**. Further inquiries can be directed to the corresponding author.

## Ethics Statement

The studies involving human participants were reviewed and approved by Except, patient written consent obtained. The patients/participants provided their written informed consent to participate in this study.

## Author Contributions

RP – first author. UG – second author. AY, DM, DK, DT, and EM – contributed equally. JP – senior authorship. All authors contributed to the article and approved the submitted version.

## Conflict of Interest

JP discloses Honoraria from Huron Consulting group and Novocure, research support from Varian Medical Systems and Kroger outside the submitted work.

The remaining authors declare that the research was conducted in the absence of any commercial or financial relationships that could be construed as a potential conflict of interest.

## Publisher’s Note

All claims expressed in this article are solely those of the authors and do not necessarily represent those of their affiliated organizations, or those of the publisher, the editors and the reviewers. Any product that may be evaluated in this article, or claim that may be made by its manufacturer, is not guaranteed or endorsed by the publisher.
