# Supplementary material for: Germline BAP1 Mutation in a Family With Multi-Generational Meningioma With Rhabdoid Features: A Case Series and Literature Review
Source: Front Oncol. 2021 Aug 24;11:721712. doi: 10.3389/fonc.2021.721712 (PMC8421801; doi:10.3389/fonc.2021.721712)
Supplement: Supplementary file 1 [file DataSheet_1.docx]

**METHODS: DNA Sequencing**

Whole exome sequencing was performed on DNA extracted from snap frozen tumor specimens and comparator peripheral blood mononuclear cells (PBMCs). Libraries were prepared with 250 ng of input DNA using NEB Ultra II FS kits (New England Biolabs). Target enrichment by hybrid capture was performed with IDT xGen Exome Research Panel v2.0 enhanced with the xGenCNV Backbone Panel and Cancer spike-in (Integrated DNA Technologies, Coralville, IA). Paired-end 151-bp reads were generated on the Illumina NovaSeq6000 instrument. Secondary analysis was performed using Churchill, a comprehensive workflow for taking raw reads from alignment through to germline and somatic variants calls [PMID: 25600152]. Reads were aligned to the human genome reference sequence (build GRCh38) using BWA (v0.7.15). Sequence alignments were refined according to community-accepted guidelines for best practices (https://www.broadinstitute.org/gatk/guide/best-practices). Germline variants were called using GATK’s HaplotypeCaller. Somatic single nucleotide variation (SNV) and indel detection were performed using MuTect-2 [PMID: 23396013]. Somatic copy number alteration (CNA) and loss of heterozygosity (LOH) were assessed using VarScan2 [PMID: 22300766].
