# Supplementary figures and images for: Germline BAP1 Mutation in a Family With Multi-Generational Meningioma With Rhabdoid Features: A Case Series and Literature Review
Source: Front Oncol. 2021 Aug 24;11:721712. doi: 10.3389/fonc.2021.721712 (PMC8421801; doi:10.3389/fonc.2021.721712)

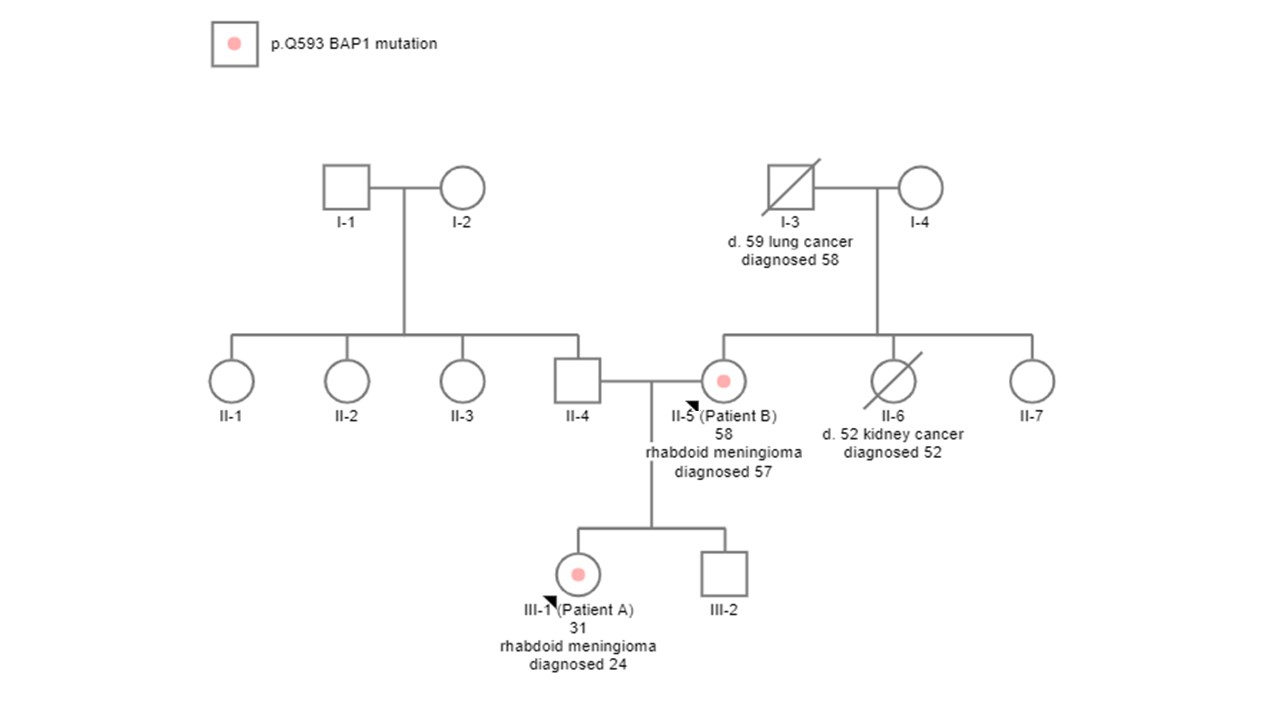

Supplement: Supplementary file 2 [file Image_1.jpeg]
